# High Culturable Bacterial Diversity From a European Desert: The Tabernas Desert

**DOI:** 10.3389/fmicb.2020.583120

**Published:** 2021-01-08

**Authors:** Esther Molina-Menor, Helena Gimeno-Valero, Javier Pascual, Juli Peretó, Manuel Porcar

**Affiliations:** ^1^Institute for Integrative Systems Biology I^2^SysBio (University of València-CSIC), Paterna, Spain; ^2^Darwin Bioprospecting Excellence S.L., Parc Científic Universitat de València, Paterna, Spain; ^3^Departament de Bioquímica i Biologia Molecular, Universitat de València, Burjassot, Spain

**Keywords:** microbial diversity, Tabernas Desert, drylands ecology, biocrust, Actinobacteria

## Abstract

One of the most diverse ecological niches for microbial bioprospecting is soil, including that of drylands. Drylands are one of the most abundant biomes on Earth, but extreme cases, such as deserts, are considered very rare in Europe. The so-called Tabernas Desert is one of the few examples of a desert area in continental Europe, and although some microbial studies have been performed on this region, a comprehensive strategy to maximize the isolation of environmental bacteria has not been conducted to date. We report here a culturomics approach to study the bacterial diversity of this dryland by using a simple strategy consisting of combining different media, using serial dilutions of the nutrients, and using extended incubation times. With this strategy, we were able to set a large (254 strains) collection of bacteria, the majority of which (93%) were identified through 16S ribosomal RNA (rRNA) gene amplification and sequencing. A significant fraction of the collection consisted of Actinobacteria and Proteobacteria, as well as Firmicutes strains. Among the 254 isolates, 37 different genera were represented, and a high number of possible new taxa were identified (31%), of which, three new *Kineococcus* species. Moreover, 5 out of the 13 genera represented by one isolate were also possible new species. Specifically, the sequences of 80 isolates held a percentage of identity below the 98.7% threshold considered for potentially new species. These strains belonged to 20 genera. Our results reveal a clear link between medium dilution and isolation of new species, highlight the unexploited bacterial biodiversity of the Tabernas Desert, and evidence the potential of simple strategies to yield surprisingly large numbers of diverse, previously unreported, bacterial strains and species.

## Introduction

Only a small fraction of the microbial diversity of our planet can be cultured under traditional microbiological tools, media, and strategies (Overmann et al., 2017; Steen et al., 2019). The use of ribosomal RNA (rRNA) genes and other genomic sequences as key markers in microbial ecology led to the discovery of a previously unknown microbial diversity (Rappé and Giovannoni, 2003). Traditional culturing techniques have allowed the isolation of rapidly-growing, easy-to-culture microbial taxa, thus relegating the vast majority of the microbial world to the imprecise group of “unculturable” organisms, which has been addressed as the “microbial dark matter” (Bernard et al., 2018; Bowman, 2018).

The generally accepted “1% culturability paradigm,” which refers to the fact that around 99% of environmental microorganisms are in fact unculturable, was recently revised by Martiny (2019). The evidence based on the study of different environments no longer supports some of the interpretations. However, even though this “1%” is probably no longer acceptable, as discussed by Steen et al. (2019), microbiologists are still far from culturing most of the existing bacterial diversity.

One of the reasons behind the obstacles in culturing environmental bacteria is the difficulty in mimicking the particular environmental conditions that certain microbes require for growth, as standard laboratory conditions may differ from the natural habitats (Stewart, 2012). Small variations in a wide variety of parameters make almost impossible to cover the immense number of different micro-environments that can be found in nature. Moreover, not only abiotic factors but also the interactions between species and metabolic cooperation are critical for the development of specific taxa that may be reluctant to grow under laboratory conditions (Vartoukian et al., 2010).

Multi-omic data have broadened our knowledge on previously unknown microbial communities and taxa (Vilanova and Porcar, 2016). Specifically, metataxonomics, which refers to the high-throughput characterization of the microbiota emphasizing on the taxonomic status and relationships between taxa (Marchesi and Ravel, 2015), has shed light on the actual composition of microbial communities, whereas metagenomics has partially unveiled some key ecological interactions, with no need for cultivating microorganisms (Lok, 2015).

Metagenomic data have been used in order to design tailor-made culture media for specific samples (Gutleben et al., 2020), but the inference of metabolic features and the functional analysis depends on the availability of complete annotated genes or genomes in public databases, what leads to failure in function assignment due to mislead annotations in genomes (Choi et al., 2016). All possible novel metabolic pathways are as diverse as the microorganisms that feature them, highlighting the countless potential applications that can derive from the uncultured microbial world (Overmann et al., 2017).

Sophisticated culture-dependent techniques have already been developed, such as the so-called ichip device, which is based on the cultivation of microorganisms by incubating the chips *in situ*, overcoming the problem of mimicking the environmental conditions (Berdi et al., 2017), or reverse genomics, which uses genomic data in order to obtain targeted antibodies to capture and sort specific cell types (Cross et al., 2019). Moreover, culturing gut microbes through techniques, such as “dilution to extinction” (Gross et al., 2015), in complete anaerobic flow work (Browne et al., 2016) and the selection of micro colonies in combination with multiple incubation conditions (Lagier et al., 2016), among other efforts in cultivating “uncultured” bacteria, have resulted in a considerable increase in the number of identified species (Martiny, 2019). Moreover, increasing the incubation times and diluting media have also proven to be useful for this purpose (Gutleben et al., 2020).

Soils are known to harbor a wide bacterial diversity with an immense potential in biotechnology and biomedicine. Actinobacteria are the major inhabitants of soil environments and one of the most ancient groups of bacteria (Battistuzzi et al., 2004), which play an important role from the ecological point of view in maintenance processes (Lewin et al., 2016). They are well-known because of their ability to produce a wide range of bioactive secondary metabolites with immense potential and applications, in particular due to their role in the synthesis of antibiotics (Zhao et al., 2018), but also enzymes, such as cellulases, which can be used in the industrial-scale breakdown of cellulosic plant biomass into simple sugars that can then be converted into biofuels (Lewin et al., 2016).

Among the different terrestrial ecosystems and soil environments, drylands have been discovered as a source of biotechnologically-relevant bacterial strains (Azua-Bustos and González-Silva, 2014; Mohammadipanah and Wink, 2016). Extreme environments, such as deserts, have been found to host a wide diversity of microbial taxa adapted to live under such harsh conditions of temperature, desiccation, and radiation, among which low water and nutrient availability are the main limiting factors for organisms (Saul-Tcherkas et al., 2013). These factors shape the local biocenosis and act as a selection pressure toward interesting mechanisms to overcome the hurdles of living in an extreme ecological niche. Both culture-dependent and -independent studies have been performed in the Atacama, the Sahara, or the Gibson deserts (Lester et al., 2007; Azua-Bustos et al., 2014; Belov et al., 2018). However, European arid lands have been poorly studied from both points of view.

The Tabernas Desert, located in the province of Almeria (southeastern Spain), is a particular, not very much studied region to date in terms of bacterial culturability. It is formed by marls, sandstone, and scarce vegetation. The annual rainfall is below 250 mm, its climate is considered hot semiarid to desert climate, depending on the altitude, and it has interesting, well-developed biocrusts.

Biological soil crusts are multi-organism consortia worldwide—spread in dryland landscapes, being estimated that around 12% of the total terrestrial surface is covered by biocrust (Rodríguez-Caballero et al., 2018). Specifically, Miralles et al. (2020) recently reported how biocrusts influenced the microbial communities in the surrounding soil, which also depend on multiple environmental factors, such as pH, temperature, or salinity, and other biotic interactions. Moreover, Maestre et al. (2013) have studied in detail the effect of climate change on the microbial biocenosis associated with biocrust in different areas, including Sorbas, which is in the vicinities of the Tabernas Desert. The studies revealed that changes in biocrusts play a major role in further modeling the composition of microorganisms.

In the present work, we have characterized the microbial communities of the Tabernas Desert by using improved culturomics techniques that have allowed the identification of an unprecedented rich diversity of bacterial strains from this unique European dryland. We hypothesized that by combining culture laboratory techniques and increasing incubation times, a higher diversity of bacterial taxa would be isolated. The results obtained confirm that simple strategies in culturomics can lead to a better understanding of the culturable fraction of microbial communities, including the isolation of new and potentially new microbial taxa.

## Materials and Methods

### Sample Collection

**FIGURE 1:**
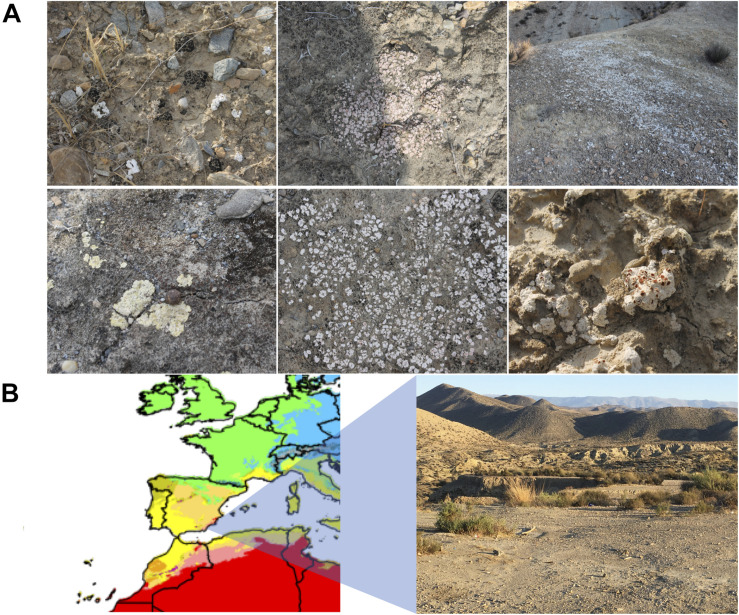
**(A)** Biocrust sampling sites in the Tabernas Desert. Six different biocrusts are shown. **(B)** Climate map zoom of Spain in the Köppen–Geiger climate classification map (1980–2016) from Beck et al. (2018). The different climate areas are represented in colors. Warm-arid regions, corresponding to desert environments, are represented in red. Picture of the sampling area in the vicinity of the natural Park of the Tabernas Desert (Almeria, southeastern Spain).

Biocrust samples were obtained in September 2018 at the Tabernas Desert, in the vicinity of the Natural Park. Sampling was carried out by taking in Falcon tubes the upper 1–2 cm of biocrust-covered surfaces in three different locations (geolocation 1: 37.0083240, −2.4532390; geolocation 2: 37.0215300, −2.4323020; geolocation 8: 37.0226290, −2.4295450), and a total of eight samples were collected of different biocrusts in terms of color and appearance (Figure 1), which were further used for culturing assays. Sample 1.1.1 corresponded to a black stained biocrust; sample 1.2.1 was from a white-pink biocrust; samples 1.3.1, 2.1.1, and 8.1.1 were all white biocrust with similar appearance; sample 1.4.1 was a yellowish biocrust; sample 2.5.1 was a white smooth biocrust; and sample 8.3.1 was a dark yellow biocrust (Table 1).

**TABLE 1:** List of biocrust samples.

	Sample	Appearance
Geolocation 1	1.1.1	Black
	1.2.1	White-pink
	1.3.1	White
	1.4.1	Pale yellow
Geolocation 2	2.1.1	White
	2.5.1	White smooth
Geolocation 8	8.1.1	White
	8.3.1	Dark yellow

### Isolation of Bacterial Strains

The samples were homogenized by mixing 1 g of biocrust with 1 ml of sterile phosphate buffer saline (PBS) 1×. Then, serial dilutions of the suspensions were prepared, and 50 μl of the 10^–3^–10^–5^ samples was spread on Petri dishes containing either Tryptic Soy Agar (TSA) medium (composition in g/L: 15.0 tryptone, 5.0 soya peptone, 5.0 sodium chloride, 15.0 agar) or Reasoner’s 2A (R2A) medium (composition in g/L: 1 peptone, 0.5 yeast extract, 0.5 dextrose, 0.5 soluble starch, 0.3 dipotassium phosphate, 0.05 magnesium phosphate, 0.3 sodium pyruvate, 15.0 agar) at concentrations 1 × (standard concentration), 0.1×, and 0.01 × (10 and 100 times diluted, respectively). TSA is a rich media used for general purposes, whereas R2A is a nutrient-poor media that favors the isolation of slow-growing and oligotrophic bacteria. Media were sterilized by autoclaving at 121°C for 20 min. Agar was autoclaved separately to the nutrient solution of each medium and added just before pouring the media into the plates. After 2 weeks of incubation at room temperature (23°C), individual colonies were selected based on their color and morphology and isolated by independent re-streaking on fresh media in order to obtain them in pure culture. New colonies were also selected a month of incubation, in order to identify, small, slow-growing microorganisms. A total of 254 isolates were cryo-preserved as a glycerol stock (20% glycerol in PBS, vol:vol) at −80°C until required.

### Colony Identification Through 16S rRNA Sequencing

A loopful of 3 μl of microbial biomass from grown plates was suspended in 100 μl of Milli-Q sterile water and then boiled for 10 min before the PCR in order to ensure cellular lysis. Colony PCR was used for taxonomic identification through 16S rRNA gene sequencing by using the universal primers 8F (5′-AGAGTTTGATCCTGGCTCAG-3′) (Edwards et al., 1989) and 1492R (5′-GGTTACCTTGTTACGACTT-3′) (Stackebrandt and Liesack, 1993). PCR was carried out with a step of incubation at 94°C for 5 min, then 24 cycles of denaturation at 94°C for 15 s, annealing at 48°C for 15 s, elongation at 72°C for 5 min, and a final elongation step at 72°C for 5 min.

Amplifications were visualized by electrophoresis in a 1.2% agarose gel stained with GelRed nucleic acid gel stain (Biotium, CA, United States) (100 V for 30 min). Amplicons were precipitated overnight in isopropanol 1:1 (vol:vol) and potassium acetate 1:10 (vol:vol) (3 M, pH 5) at −20°C. DNA was pelleted by centrifugation at 12,000 rpm for 10 min, then washed with ethanol 70%, and resuspended in the required 15 μl of Milli-Q water. BigDye^®^ Terminator v3.1 Cycle Sequencing Kit (Applied Biosystems, Carlsbad, CA, United States) was used to tag amplicons, which were sequenced with the Sanger method by the Sequencing Service (Servei Central de Suport a la Investigació Experimental SCSIE) of the University of Valencia (Spain). All sequences were manually edited with Trev (Bonfield and Staden, 2002) to eliminate low-quality base calls, and final sequences were compared by EzBioCloud 16S identification BLAST tool to nucleotide databases. Primers 341R (5′-CTGCTGCCTCCCGTAGG-3′) (Muyzer et al., 1996) and 1055F (5′-ATGGCTGTCGTCAGCT-3′) (Harms et al., 2003) were used for whole 16S rRNA sequencing in order to fully identify the isolates holding an identity lower than 98.7% with the closest type strain by sequencing partially the 16S rRNA gene. The MEGA7 tool was used to assemble the whole 16S rRNA gene sequence. The quality of the chromatograms was checked with the Sequence Scanner software. The quality value (QV) threshold was 20. In the case of competing peaks, the highest peak in both forward and reverse sequences after the assembly was chosen. The sequences were manually revised afterward and have been deposited under the GenBank/EMBL/DDBJ accession numbers MT749781–MT750013 and MN069869–MN069868.

In order to analyze the closest environmental clone or isolates of our strains, an extensive blast was carried out against the NCBI Nucleotide collection (nr/nt) databases optimized for highly similar sequences.

## Results

### Isolation of Bacterial Strains

**FIGURE 2:**
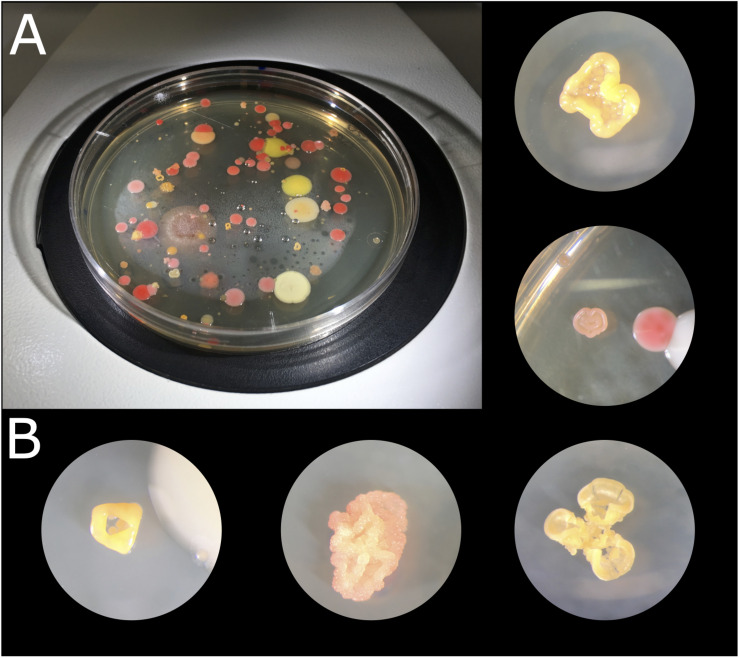
Microbial colonies obtained after culturing biocrust samples at 23°C for 2 weeks. **(A)** TSA 1× plate of sample 1.4.1. **(B)** Microbial colonies under the binocular loupe.

Culturing different biocrust samples from the Tabernas Desert yielded a large number of different colonies in terms of color, shape, and morphology (Figure 2). A total of 254 strains were isolated in pure culture, which were named as T or R depending on the medium from which they had been isolated (TSA or R2A, respectively) and numbered consecutively. There was no significant fungal growth, and most of the bacterial colonies displayed bright colors, being the most abundant those of red, pink, orange, and yellow-pigmented bacteria. There were also dark stained isolates, and some others changed from orange to purple with time. The morphology of the isolates was also diverse, being particularly curious those growing in 3D structures and cell clumps (Figure 2B). Isolates T1 to T159 were isolated from TSA plates after 2 weeks of incubation, of which 131 were obtained in pure culture and cryopreserved for further use; isolates R1 to R115 were selected from R2A plates, of which only 76 were obtained in pure culture. It was not possible to obtain all the selected colonies in pure culture due to crossed contamination or growth failure after several re-streaking steps. After a month of incubation of the plates, small colonies were also selected, and 47 of them were obtained in pure culture, being 20 of them originally from TSA and 27 from R2A plates.

### Colony Identification Through 16S rRNA Sequencing

**FIGURE 3:**
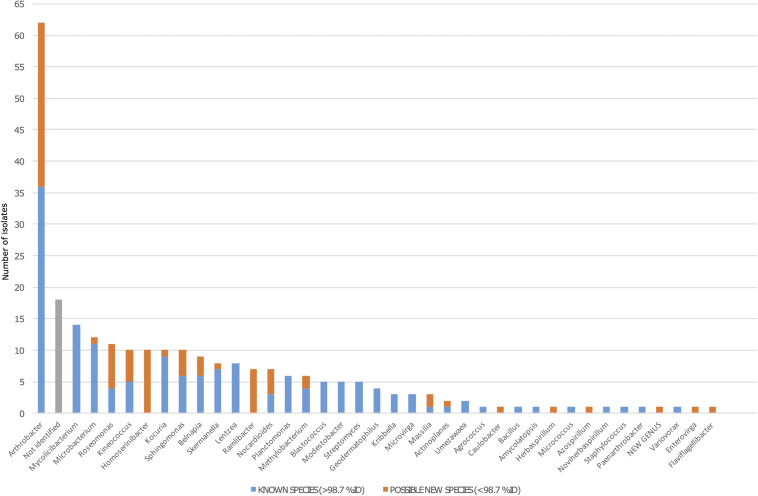
Bacterial diversity among the 254 isolates from the Tabernas Desert collection. The blue bar represents the number of fully identified isolates in each genus (>98.7% of identity against 16S rRNA databases). The orange bar represents the number of possible new species in each genus (as defined by <98.7% of identity against 16S rRNA databases). Not identified isolates are shown in gray.

From the 254-strains collection, a total of 236 isolates (93%) were identified, belonging to 37 different genera, and only 18 isolates remained not identified due to either a lack of amplification in PCR or failure in sequencing. A total of 62 isolates were identified as *Arthrobacter* spp., which was the most abundant genus representing 24.8% of the collection. The second most represented genus was *Mycolicibacterium*, holding a 7.09% of abundance, similar to the abundances of *Microbacterium* and *Roseomonas*. In contrast, the less abundant genera were *Agrococcus*, *Amycolatopsis*, *Azospirillum*, *Bacillus*, *Caulobacter*, *Enterovirga*, *Flaviflagellibacter*, *Herbaspirillum*, *Micrococcus*, *Noviherbaspirillum*, *Paenarthrobacter*, *Staphylococcus*, and *Variovorax*, being represented only by one isolate each (Figure 3).

A total of 80 isolates in the collection showed a percentage of identity lower than 98.7%, suggesting that they could represent new species within that genus (Chun et al., 2018). Out of the 80 possible new species, 26 isolates were identified as *Arthrobacter* spp., whereas the rest were distributed among half of the genera detected. Interestingly, between the genera that had at least one possible new species, five of them were represented by just one isolate (*Caulobacter*, *Herbaspirillum*, *Azospirillum*, *Enterovirga*, and *Flaviflagellibacter*) (Figure 3). For example, we isolated five *Kineococcus* strains as being possible new species, and three were further identified and described. The three new species have recently been published with valid names *Kineococcus vitellinus* sp. nov., *Kineococcus indalonis* sp. nov., and *Kineococcus siccus* sp. nov. (Molina-Menor et al., 2020). Moreover, the isolates identified as *Homoserinibacter* and *Ramlibacter*, which were 10 and 7, respectively, were all potential new species, and one isolate in the collection showed an ID value lower than 95%, which is the threshold value for new genus description (Chun et al., 2018). This possible new genus, represented by the isolate named as T16, would be closely related to *Roseomonas*, within the Acetobacteraceae family. Other genera identified in the collection were *Actinoplanes*, *Belnapia*, *Blastococcus*, *Geodermatophilus*, *Kocuria*, *Kribbella*, *Lentzea*, *Massilia*, *Methylobacterium*, *Microvirga*, *Modestobacter*, *Nocardioides*, *Planctomonas*, *Skermanella*, *Sphingomonas*, *Streptomyces*, and *Umezawaea* (Figure 3).

**FIGURE 4:**
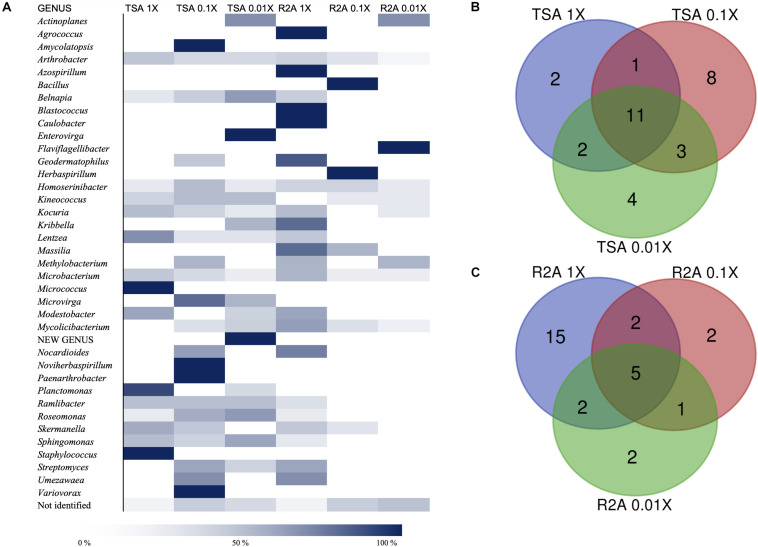
Taxonomic diversity as a function of the culture media and dilutions used. **(A)** Heatmap of relative abundances of the different genera, possible new genera, and non-identified isolates among media (TSA at 1, 0.1, and 0.01×; R2A at 1, 0.1, and 0.01×). The genera are listed in alphabetical order. Possible new genera and non-identified isolates are included. **(B)** Venn diagram of shared and exclusive taxa at the genus level in TSA media at three different concentrations (1, 0.1, and 0.01×). Possible new genera and non-identified groups are included. **(C)** Venn diagram of shared and exclusive taxa at the genus level in R2A media at three different concentrations (1, 0.1, and 0.01×). Possible new genera and non-identified groups are included.

As for the isolation conditions, it was possible to analyze the bacterial diversity according to the media from which the genera had been isolated, as well as the incubation time required for their isolation. Almost 60% of the genera were isolated from at least two different media (TSA or R2A at three concentrations), whereas 15 genera were isolated just from one of the tested conditions (Supplementary Table 1). The quantitative distribution of genera among the different isolation media is shown in Figure 4A. In the case of *Arthrobacter* and *Microbacterium*, isolates were identified in all six media but, most frequently, in the most concentrated ones. Others, such as *Actinoplanes*, were isolated from the most diluted concentration on TSA and R2A, and the possible new genus was isolated from TSA 0.01×. Non-identified strains were also isolated from all six media, although the higher abundance was detected in the less concentrated ones, especially on R2A 0.01×. Interestingly, the five *Blastococcus* isolates grew on R2A 1× (Figure 4A).

**FIGURE 5:**
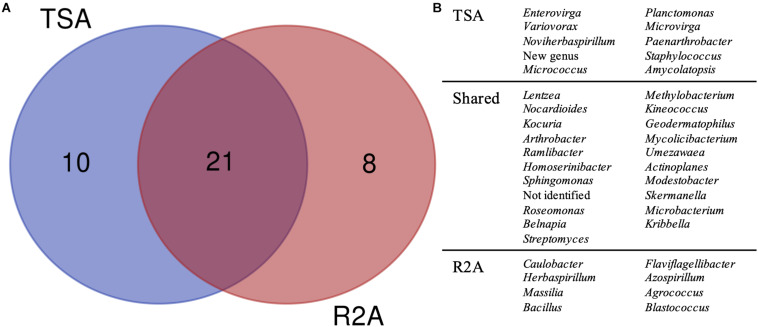
Taxonomic diversity as a function of the culture media and dilutions used. **(A)** Venn diagram comparing the genera isolated in TSA medium and R2A medium, considering all the three dilutions used for microbial growth (1, 0.1, and 0.01×). Possible new genera and non-identified groups are included. **(B)** List of genera represented in the Venn diagram.

The co-isolation of genera in the different nutrient conditions was compared through Venn diagram representations, which are shown in Figures 4B,C, listed in Tables 2, 3, and in Figure 5. In the case of TSA media, 11 different genera were detected in the three concentrations assayed. The concentration that allowed the isolation of more exclusive taxa was the intermediate 0.1×, with eight unique taxa, in contrast with two on TSA 1× and four on TSA 0.01× (Figure 4B). On the contrary, for R2A media, the concentrated R2A 1× seemed the best media for isolating different genera, being only five of them common for the three concentrations. Only two exclusive taxa were isolated from 0.1 and 0.01 × R2A (Figure 4C). In general, thus, bacterial genera tended to be shared by the TSA media, regardless of the dilution. In contrast, genera isolated from R2A tended to concentrate in the non-diluted medium.

**TABLE 2:** List of coincident genera in the TSA media as it is compared in the Venn diagram in Figure 4B.

Media	N.	Genera
TSA 1×, TSA 0.1×, TSA 0.01×	11	*Lentzea*, *Belnapia*, *Kocuria*, *Arthrobacter*, *Ramlibacter*, *Homoserinibacter*, *Sphingomonas*, *Roseomonas*, *Kineococcus*, *Microbacterium*, not identified
TSA 1×, TSA 0.1×	1	*Skermanella*
TSA 0.1×, TSA 0.01×	3	*Streptomyces*, *Mycolicibacterium*, *Microvirga*
TSA 1×, TSA 0.01×	2	*Planctomonas*, *Modestobacter*
TSA 1×	2	*Micrococcus*, *Staphylococcus*
TSA 0.1×	8	*Methylobacterium*, *Geodermatophilus*, *Nocardioides*, *Variovorax*, *Umezawaea*, *Amycolatopsis*, *Paenarthrobacter*, *Noviherbaspirillum*
TSA 0.01×	4	New genus, *Enterovirga*, *Actinoplanes*, *Kribbella*

**TABLE 3:** List of coincident genera in the R2A media as it is compared in the Venn diagram in Figure 4C.

Media	N.	Genera
R2A 1×, R2A 0.1×, R2A 0.01×	5	*Mycolicibacterium*, *Arthrobacter*, *Homoserinibacter*, *Microbacterium*, not identified
R2A 1×, R2A 0.1×	2	*Skermanella*, *Massilia*
R2A 0.1×, R2A 0.01×	1	*Kineococcus*
R2A 1×, R2A 0.01×	2	*Kocuria*, *Methylobacterium*
R2A 1×	15	*Lentzea*, *Agrococcus*, *Umezawaea*, *Nocardioides*, *Geodermatophilus*, *Blastococcus*, *Modestobacter*, *Curvibacter*, *Sphingomonas*, *Caulobacter*, *Roseomonas*, *Azospirillum*, *Belnapia*, *Streptomyces*, *Kribbella*
R2A 0.1×	2	*Bacillus*, *Herbaspirillum*
R2A 0.01×	2	*Actinoplanes*, *Flaviflagellibacter*

The comparison, regardless of the dilution factor, of TSA and R2A media in terms of genera diversity revealed that one fourth of the groups (as non-identified isolates and the possible new genus were included) had been exclusively isolated in TSA, whereas around 20% were unique taxa for R2A (Figure 5). This revealed a clear preference of some genera to grow under specific environments, as almost half of them were only isolated from one of the media used. Interestingly, all the genera that were isolated exclusively in TSA were represented by one isolate, except for *Planctomonas*, which was represented by six (five from TSA 1× and one from TSA 0.01×). In the case of the unique R2A taxa, apart from the five *Blastococcus* and three *Massilia* strains, the rest were also represented by one isolate.

**FIGURE 6:**
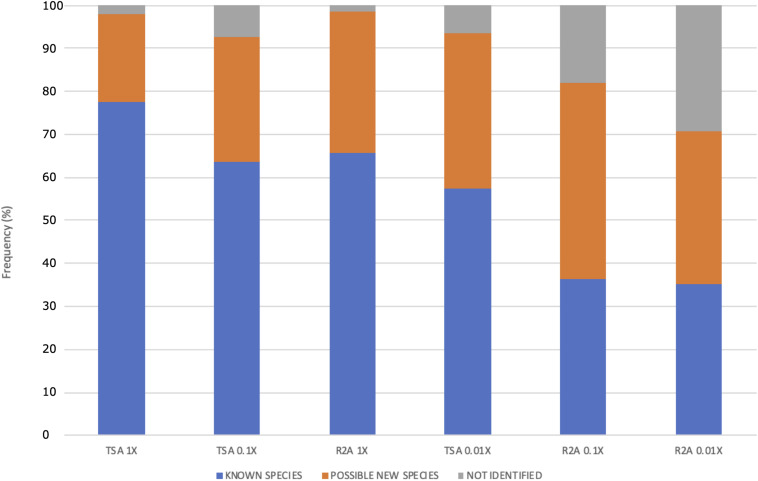
Frequency (%) of possible new species, known species, and non-identified isolates as function of the culture media and the dilution used. The media are ordered according to the concentration of protein hydrolysate. Two different media, TSA and R2A, were used at three different concentrations: 1, 0.1, and 0.01×.

Moreover, the distribution of the possible new species and the non-identified isolates was different among the media dilution used (Figure 6). The ones that gave the higher fraction of potential new taxa were the lowest nutrient concentrations (media TSA 0.1 and 0.01× and all the R2A combinations). Specifically, the best results for new taxa isolation were obtained in R2A 0.1×, whereas the highest fraction of non-identified ones was obtained in R2A 0.01×. Interestingly, the sum of both groups of isolates (potential new species and unidentified ones) comprised a similar percentage in these two media, being almost 65%, which is approximately a threefold increase with respect to the TSA 1× group.

**FIGURE 7:**
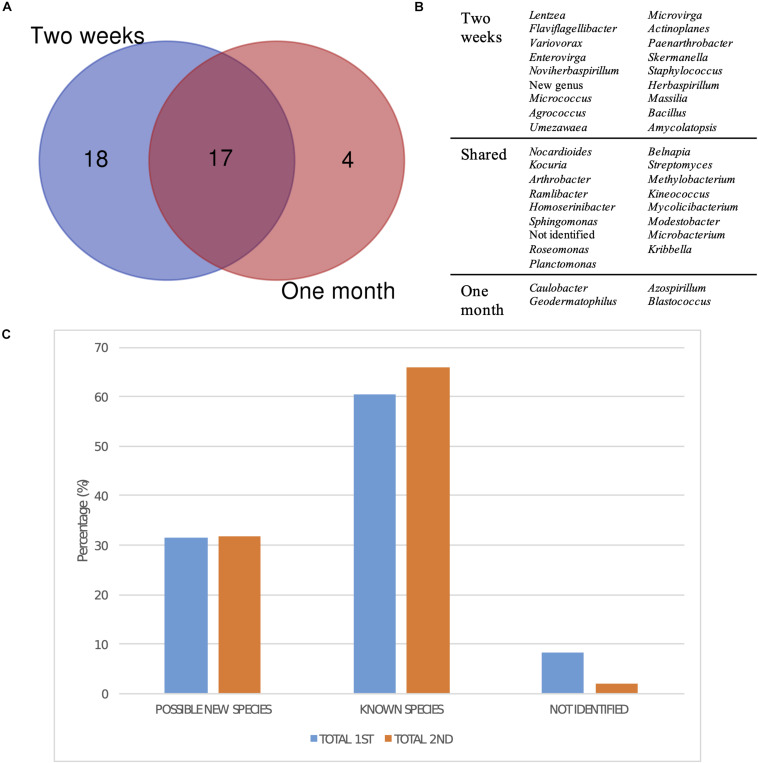
Taxonomic diversity as a function of isolation date. **(A)** Venn diagram comparing the genera isolated after 2 weeks and a month of incubation. New genus and non-identified groups are included. **(B)** List of genera represented in the Venn diagram. **(C)** Bar plot representing the percentage of possible new taxa, known taxa, and non-identified isolates at the genus level after 2 weeks (1st selection) and a month of incubation (2nd selection).

Regarding the differences in diversity depending on the incubation time after which each bacterial strain was isolated, the comparison of the groups, at the genus level, isolated after 2 weeks and after 1 month of incubation revealed that most of the taxa had been already detected in the first selection (Figure 7A). However, four genera were exclusively isolated after 1 month, which were *Caulobacter*, *Geodermatophilus*, *Azospirillum*, and *Blastococcus* (Figure 7B). Moreover, there was no significant difference in isolating potentially new taxa, as the percentage of isolates showing an identity percentage below 98.7 with the closest blast was similar between both selection times.

Finally, the differences in diversity observed depending on the biocrust sample and sampling site were analyzed (Supplementary Tables 2–4). The geolocation that yielded the highest number of colonies was geolocation 1, with as much as 60% of the total isolates. On the contrary, geolocations 2 and 3 yielded a total of 51 isolates each. In terms of number of different genera identified, the most diverse sample was 1.4.1, from which 23 genera were isolated in pure culture. Although sample 1.4.1 was also the one that yielded the highest number of colonies, 30% of them corresponded to *Arthrobacter* spp.

*Arthrobacter* spp., which was the most abundant genus, was isolated from seven out of the eight samples, with different abundances. Particularly, in sample 2.1.1, half of the isolates belonged to this genus. Interestingly, 8 of the 10 *Homoserinibacter* spp. were from sample 1.4.1, but none had been isolated from geolocations 2 and 3, and the *Kineococcus* genus was also unique for the four samples of geolocation 1.

### Ecological Novelty

Many of our strains were not phylogenetically related with any previously cultured strain. Specifically, 10.6% of the isolates shared less than 98.7% of 16S rRNA gene sequence similarity against any isolate previously studied, and 5.5% of the strains shared less than 98.7% regarding any environmental clone. Moreover, 24.8% of our isolates showed similarities with, to date, uncultured microorganisms (Supplementary Table 5).

Up to 40.5% of the closest neighbors of our strains were environmental clones or isolates associated with soil environments, being 4.7% of them specifically from deserts around the globe, such as the Mojave Desert (Southwestern United States) and the Badain Jaran Desert (China), among others. Others (8.66%) were closely linked to clones or isolates inhabiting extreme environments, such as oil or heavy metal-contaminated soils, or cold environments, such as glaciers and permafrost samples. Furthermore, 8.3% of our strains were related to environmental clones or isolates found in aquatic habitats, mainly rainwater but also lakes, fish ponds, and freshwater sediments. Interestingly, 8.7% of the closest relatives corresponded to plant-associated clones, either associated with the rhizosphere, phyllosphere, or endophytic bacteria, and a small fraction representing 4.7% of the collection was similar to animal-associated bacteria, such as the gut of insects or corals.

## Discussion

### The Tabernas Desert Soils Harbor a Wide Bacterial Diversity

Although drylands and cold and hot deserts are a source of biotechnologically-relevant bacterial strains (Azua-Bustos and González-Silva, 2014; Mohammadipanah and Wink, 2016), the unique ecosystem of the Tabernas Desert remained poorly unexplored from the culturomics point of view. In the present study, we established a large collection consisting of, at least, 254 bacterial strains from 37 genera, in addition to one possible new genus, which belonged to three phyla.

The distribution of phyla in the collection resembles the microbial communities studied in other desert environments, such as the Atacama, the Gibson, or the Sahara Desert, with Actinobacteria as the most abundant phylum, followed by Proteobacteria and, to less extent, Firmicutes, among the culturable fraction (Belov et al., 2018; Schulze-Makuch et al., 2018). These taxa are also the ones previously detected in airborne sand particles from the Sahara Desert (Meola et al., 2015). Moreover, 19 different families were identified in the present work within those phyla: ten Actinobacteria families, five α-Proteobacteria, two β-Proteobacteria, and two Firmicutes. Actinobacteria, as reported by many authors, are one of the most common inhabitants in soil, but also particularly abundant in what is considered the extremobiosphere (Bull, 2011; Mohammadipanah and Wink, 2016).

The role of Actinobacteria in the synthesis of bioactive microbial metabolites with high pharmacological and commercial interests, such as antibiotics, is well-known. In particular, *Microbacterium* and *Lentzea* isolates, as well as other represented genus to lesser extent, such as *Streptomyces*, *Amycolatopsis*, and *Actinoplanes*, may be playing a role in the modulation of communities through competitors’ growth inhibition (Tao et al., 2016; Zhao et al., 2018). Nithya et al. (2015) reported the isolation of 16 actinobacterial strains from the desert in Arabia Saudi that exhibited antimicrobial potential, and Hoshino et al. (2018) described for the first time a new type of macrolactams named as umezawamides as they are synthetized by the genus *Umezawaea*. Moreover, Wichner et al. (2017) reported the isolation of six new metabolites that had never been traced before from microbial sources by a novel strain of *Lentzea* sp. from the Atacama Desert, which confirms the potential that these bacteria hold in the synthesis of interesting secondary metabolites.

Other groups within the Actinobacteria clade have been described as important complex organic matter-degrading bacteria, such as Actinomycetales, which are proficient degraders of polysaccharide of plant, animal, or fungal origin in soils. This polysaccharide degradation has a crucial impact in the carbon sources reuse cycles (Yeager et al., 2017). Glucosidases, glycoside hydrolases, carbohydrate esterases, and other related enzymatic activities are abundant in the analyzed genomes of the three new *Kineococcus* strains described from Tabernas indeed (Molina-Menor et al., 2020).

Actinobacterial species can play also an important role in bioremediation. The ability to degrade recalcitrant compounds and other environmental contaminants, such as heavy metals, has been reported for species within the Actinomycetales clade, what would have an application in the bioconversion of wastes into high-value products (Masuda et al., 2012; Inoue et al., 2016; Alvarez et al., 2017; Herrero et al., 2018). *Kocuria* and *Microbacterium* species isolated from rhizosphere have been used for the bioremediation of pesticides, such as lindane (Abhilash et al., 2011), and *Microbacterium* spp. have also been found to potentially degrade organophosphorus pesticides and polycyclic aromatic hydrocarbons (PAHs) (Panneerselvan et al., 2018). Moreover, some of the *Roseomonas* species have been first isolated from oil-contaminated soils (Chaudhary and Kim, 2017; Subhash and Lee, 2018), and *Arthrobacter* species have demonstrated their potential to degrade polycyclic aromatic compounds (Gil et al., 2000; Muangchinda et al., 2017), which is interesting as they are one of the main inhabitants of soil environments (Mongodin et al., 2006).

In fact, *Arthrobacter* is the most abundant taxa in the collection established from the Tabernas Desert, with as many as 62 strains assigned, 26 of which being possible new species. *Arthrobacter* spp. do not only play a major role in the maintenance of natural processes in soils, such as the degradation of organic matter (Yeager et al., 2017) or the recycling of different carbon sources (Adams et al., 2011; Anderson et al., 2012; Berlemont and Martiny, 2013), but also in their recovery after fires (Fernández-González et al., 2017) and in the rhizosphere as common plant growth-promoting bacteria, preventing other bacteria and nematodes infections (Topalovic et al., 2020). They are common inhabitants of extreme environments, such as warm or cold arid deserts, and the robustness of this genus makes it interesting from the biotechnological point of view as it can be a source of bioactive compounds, such as the highly valued carotenoids (Fong et al., 2001). This, summed up to the diversity that has been found in the collection, opens up possible new biotechnological applications in several areas for the isolates within this genus.

The samples from the Tabernas Desert display a wide range of colors, probably due to the presence of these carotenoids, which may be playing a major role in their natural protection against oxidative stress and other consequences of sun exposure. The relationship between the synthesis of carotenoids, among other pigments, and the resistance to UV radiation in highly irradiated environments has been well described (Villareal et al., 2016; Peyrat et al., 2019). Not only *Arthrobacter* but also the genus *Microbacterium*, the second most abundant one with 14 representatives, is also known for its ability to synthetize carotenoids. Han et al. (2016) and Reis-Mansur et al. (2019) reported the UV resistance of two carotenoid-producing *Microbacterium* spp., both isolated from Antarctica. Furthermore, the genus *Methylobacterium*, represented by six isolates in the collection, has been described to synthetize pink carotenoids, which presumably act as antioxidants against reactive oxygen species (ROS) generated by UVA and UVB stresses (Sundin et al., 2002; Gao and Garcia-Pichel, 2011). Moreover, Yoshida et al. (2017) described an avobenzone-like compound isolated from several strains within the genus *Methylobacterium* with UVA-absorption and photostability activities, whose similarity to avobenzone supports its potential use as a commercial sunscreen ingredient.

The resistance to a wide range of stressors, particularly the resistance to gamma or UV radiation, has been largely described for the genera identified in the collection. Species within the genera *Roseomonas* (Kim et al., 2018), *Kocuria* (Gholami et al., 2015; Mehrabadi et al., 2016), or *Geodermatophilus* (Montero-Calasanz et al., 2013; Hezbri et al., 2016), among others, have been described as highly resistant to radiation. Moreover, *Sphingomonas* species, which represent almost 4% of the collection, have been reported to be one of the main inhabitants of solar panels. These artificial devices, exposed to the maximum sun radiation and distributed worldwide, are colonized by a stable and diverse microbial community dominated by highly resistant bacteria (Dorado-Morales et al., 2016). Interestingly, Yu et al. (2015) reported for the first time the resistance to radiation for bacteria that had been isolated from the Taklamakan Desert belonging to the genera *Nocardioides* and *Microvirga*, among others. These two genera are represented by seven and three isolates in the collection, respectively.

As expected according to the origin of the samples, most of the taxa have been previously described as soil inhabitants, with representatives having been isolated from desert environments indeed. Not only *Arthrobacter* and *Microbacterium*, as mentioned above, but also many other species identified come from similar ecological niches. *Roseomonas* and *Kineococcus* species (Yokota et al., 1993; Lee, 2009; Liu et al., 2009; Nie et al., 2012b; Ramaprasad et al., 2015; Chaudhary and Kim, 2017; Kim et al., 2017), the only species in the genus *Homoserinibacter* (Kim et al., 2014) or *Lentzea terrea*, *Lentzea soli*, and *Lentzea jiangxiensis*, among other representatives of the genus *Lentzea*, are examples of taxa that have been described as members of soil microbial communities (Li et al., 2012, 2018a,b). Moreover, other genera, such as *Skermanella*, *Belnapia*, or *Blastococcus*, which are comprised by few members, have also representatives that come from soil or desert ecosystems (Reddy et al., 2006; Zhang et al., 2015; Castro et al., 2018; Yang et al., 2019).

Although culture-independent approaches have led to the discovery of large unknown microbial diversity, these techniques can only predict to a certain extent the biotechnological potential of the identified microorganisms. The isolation of culturable, novel bacterial or fungal taxa under laboratory conditions is thus a crucial step for the development of innovative and new whole cell-based applications. For example, desert-inhabiting extremophilic bacteria have demonstrated to be useful in improving plant toleration to drought (Marasco et al., 2012; Rolli et al., 2015). Moreover, Sibanda et al. (2017) reported a culture-based screening to identify strains able to degrade some of the compounds present in carwash effluents, such as PAHs, heavy metals, and other pollutants.

Extremophilic bacteria have proven to be an excellent source of highly resistant proteins for several industries, such as in biofuel production, biorefinery, bioremediation, or the revalorization of agricultural waste products, such as lignocellulosic material (Zhu et al., 2020). However, many of these applications rely on genetic engineering techniques and synthetic biology and represent only a fraction of their possibilities.

### Simple Culture-Based Strategies Rescue Significant Fractions of Previously Unknown Bacterial Diversity

The Tabernas Desert is particularly diverse in novel bacteria. Many of the closest relatives of our strains are environmental clones that inhabit a wide range of environments, mainly terrestrial ecosystems, including extreme environments, such as hot and cold deserts. These results highlight the importance of our collection of microorganisms from an ecological point of view (Supplementary Table 5) since we have been able to access many bacteria never before studied, including through molecular tools.

Our methodology and results have, thus, important implications in terms of defining the best strategies for the discovery of new genetic variants of the soil bacteriome. We found a clearly different pattern in the two media we analyzed. Indeed, the composition in terms of nutrients and concentration is considerably different between TSA and R2A media. TSA is a rich media, which differs from the natural environments in terms of nutrient availability as these compounds are not usually abundant, whereas R2A is a poor media with limited amount of nutrients and carbon sources. TSA media seem to be equally good for yielding different isolates, according to the high number of shared taxa (Figure 4B), whereas for R2A, it is clear that 1× combination, which is already a diluted media, is a suitable concentration for microbial growth because further dilutions yielded a very low number of colonies after 1 month (Figure 4C). However, the highest number of non-identified isolates was isolated from R2A 0.01×, with as much as five isolates that represented 27.8% of them (Figure 4A).

*Arthrobacter*, *Homoserinibacter*, and *Microbacterium* were identified in all of the tested media, whereas *Actinoplanes* or *Microvirga*, among others, was only found in diluted media (Figure 4A and Supplementary Table 1). The ability of *Microvirga* species to grow on different media has been previously reported. Species within this genus are able to grow both in concentrated media, such as TSA, and in diluted ones, such as R2A (Dahal and Kim, 2017), but others, such as *Microvirga*, are unable to grow in nutrient abundance (Huq, 2018). *Nocardioides* spp., *Geodermatophilus* spp., and *Umezawaea* spp. have been identified in TSA 0.1× and R2A 1×, in which the nutrients are at the same order of magnitude, suggesting a more specific requirement, even though there are reports of the isolation and growth of species within these genera in a broader number of media (Nie et al., 2012a; Montero-Calasanz et al., 2013; Hezbri et al., 2016).

In contrast with this, the genera *Kocuria*, *Kineococcus*, and *Mycolicibacterium* were isolated from five out of the six media used. In the case of *Kineococcus* spp., there are previous reports on their isolation from a wide diversity of media and their ability to grow on them too, including diluted TSA 0.1× (Liu et al., 2009), the rich starch-casein agar medium (Duangmal et al., 2008; Li et al., 2015), or pure TSA (Xu et al., 2017), which also happens for *Mycolicibacterium* species, as reported by Nouioui et al. (2019). Moreover, Kaewkla and Franco (2013) reported a similar strategy of isolating novel culturable strains from trees by extending incubation times and using diluted media, which resulted in the isolation of a wide diversity of genera and new species, including several *Nocardioides*, *Kribbella*, and *Amycolatopsis* isolates, which have grown preferably in intermediate to low concentrations in the case of the samples from the Tabernas Desert.

Of the 39 groups, including non-identified isolates and the possible new genus, 15 were identified in just one media, which represents almost 40% of the diversity at the genus level. This confirms that the diversity observed is highly dependent on the use of a combination of culture media, which supports the idea that combining media and diluting them increase the chances of finding different and new taxa.

It is interesting to note that the potentially new genus we identified, which is closely related to the *Roseomonas* clade according to the 16S rRNA sequencing analysis, was isolated from the most diluted TSA media. Moreover, the strain R60, isolated from the lower concentration of R2A, was initially identified as another possible new genus and is now classified within the genus *Flaviflagellibacter*, as it has been recently published by Dong et al. (2019), after the beginning of this work. Thus, isolate R60 is now a possible new species in this novel genus, which includes only one species, *Flaviflagellibacter deserti*, that was first isolated from R2A media plates that had been incubated for 10 days, although it showed the ability to grow also in more concentrated media, such as TSA and LB, suggesting a flexible behavior in terms of nutrient availability for this genus.

The fact that around 25% of the taxa at the genus level were exclusively isolated in TSA and 20% in R2A suggests that even applying dilution factors to the media and increasing the incubation times, culturing samples in a selection of media is worth it in order to obtain a higher diversity (Figure 5), and that the selection of the proper media is critical for it. Moreover, only 3 out of the 18 exclusive groups, *Planctomonas* in TSA and *Massilia* and *Blastococcus* in R2A, are represented by more than one isolate, which suggests that the less abundant taxa in culture collections are the ones with more specific requirements for growth. Interestingly, the only species described within *Planctomonas* was first isolated after 4 weeks of incubation (Liu et al., 2019).

The differences observed in the distribution of potential new species depending on the isolation media suggest that low nutrient concentrations increase the chances of identifying new taxa (Figure 6), which is in accordance with the results observed in terms of diversity at the genus level (Figures 4B,C). The percentage of possible new species increases, whereas the concentration of nutrients decreases. The concentrations in media TSA 0.1× and R2A 1× as well as and the concentrations in TSA 0.01× and R2A 0.1× are in the same order of magnitude. Interestingly, the most diluted media, R2A 0.01×, was not the best one in terms of possible new taxa, but yet exhibited the highest fraction of non-identified isolates, which can at least partially correspond to new species (Figure 6). The results obtained support the idea that traditional, concentrated media have favored the isolation of well-known, specific groups of fast-growing taxa. Almost 80% of the isolated strains in TSA 1× have been already described, whereas the fraction of unknown isolates (possible new species and non-identified ones) comprises more than 60% of the total in highly diluted media (R2A 0.1 and 0.01×) (Figure 6).

Finally, the moment in which the different genera were isolated reveals that most of them could already be detected after 2 weeks of growth (Figure 7A). However, four genera were exclusively isolated after a month, confirming that extending incubation times is important in order to obtain a higher microbial diversity, especially for isolating slow-growing and oligotrophic bacteria. One of these genera was *Blastococcus*, which is in accordance with the long incubation times reported by Yang et al. (2019) for the isolation of *Blastococcus deserti* in diluted media.

Surprisingly, the fraction of possible new species was similar after 15 days and 1 month of growth, but the percentage of non-identified isolates was higher in the first sampling (Figure 7C). Song et al. (2016) studied the effect of nutrient availability and incubation times in the development of soil microbial communities, revealing that the highest bacterial diversity is found in intermediate nutrient concentrations, as copiotrophic bacteria rapidly grow in concentrated media, whereas only resistant oligotrophic strains develop in highly diluted ones, which is in accordance with the results described above. Moreover, they observed that the peak in diversity is reached at the same time regardless of the nutrient concentration, which may explain the constant fractions of known and unknown diversity discussed at two sampling times.

Taken together, our results reveal that simple culturomics approaches, such as using combinations of media, long incubation times, and extreme dilutions of the nutrients, can be useful to yield a large diverse set of culturable microbial strains if applied to biodiverse natural samples, since we were able to build a diverse collection of bacterial strains from a few samples using these simple techniques. Our findings, combined with similar efforts, can help to fill the gap between the high numbers of bacterial genetic variants identified in culture-independent next generation sequencing (NGS) studies and the–up to date–still very low fraction of culturable microorganisms. This has, well beyond microbial ecology, important biotechnological implications.

## Data Availability Statement

The datasets generated for this study can be found in online repositories. The names of the repository/repositories and accession number(s) can be found below: https://www.ncbi.nlm.nih.gov/genbank/, MT749781–MT750013 and MN069867–MN069869.

## Author Contributions

MP conceived the work. EM-M, HG-V, and MP carried out the sampling. EM-M and HG-V performed all the experiments. EM-M, HG-V, JuP, JaP, and MP analyzed the results, wrote, and approved the manuscript. All authors contributed to the article and approved the submitted version.

## Conflict of Interest

MP and JuP are founders of Darwin Bioprospecting Excellence S.L. HG-V is an employee of Darwin Bioprospecting Excellence S.L. The remaining author declares that the research was conducted in the absence of any commercial or financial relationships that could be construed as a potential conflict of interest.
