# Supplementary material for: High Culturable Bacterial Diversity From a European Desert: The Tabernas Desert
Source: Front Microbiol. 2021 Jan 8;11:583120. doi: 10.3389/fmicb.2020.583120 (PMC7821382; doi:10.3389/fmicb.2020.583120)
Supplement: Supplementary file 1 [file Presentation_1.PPTX]

## Slide 1
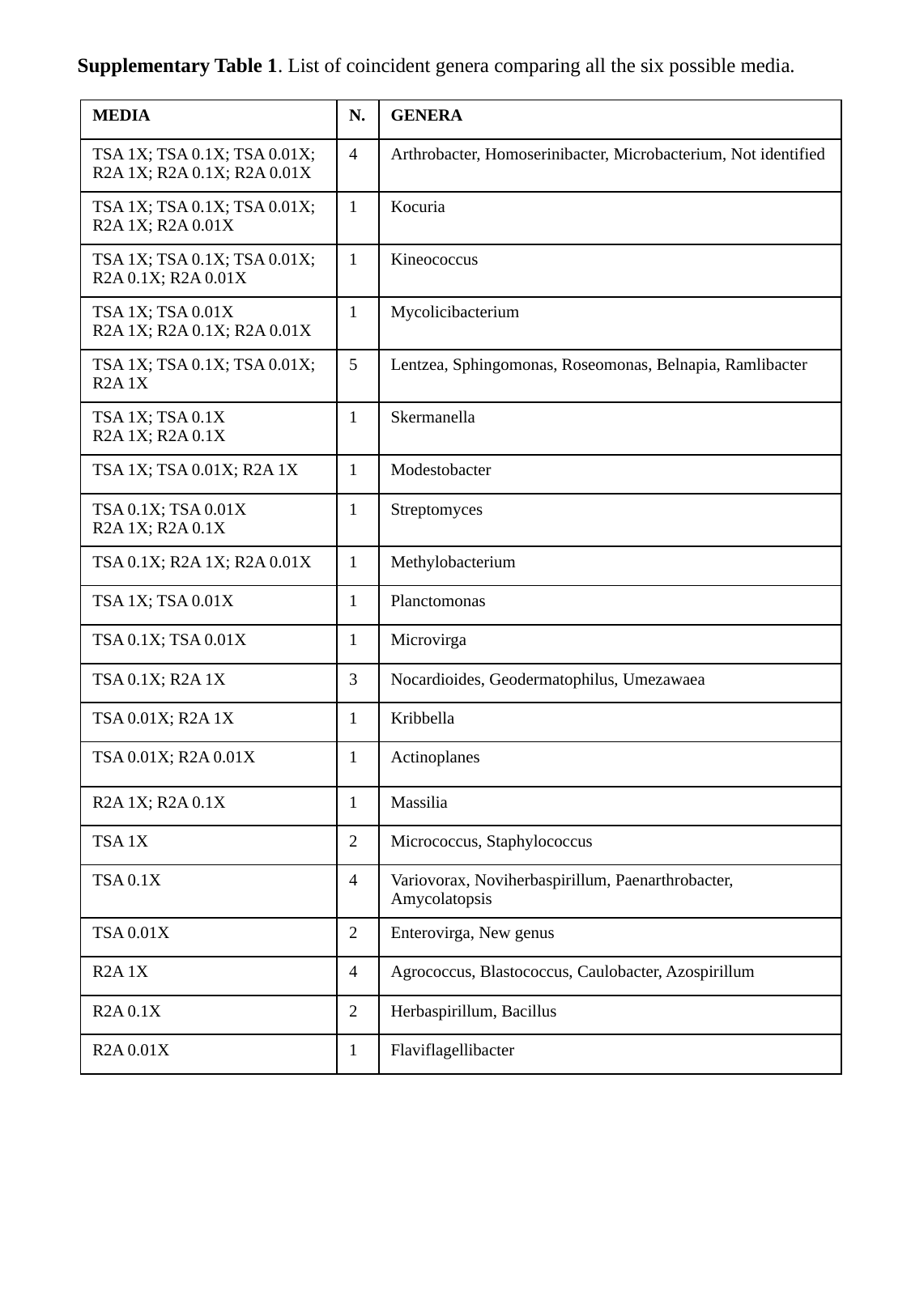

Supplementary Table 1. List of coincident genera comparing all the six possible media.
| MEDIA | N. | GENERA |
| --- | --- | --- |
| TSA 1X; TSA 0.1X; TSA 0.01X; R2A 1X; R2A 0.1X; R2A 0.01X | 4 | Arthrobacter, Homoserinibacter, Microbacterium, Not identified |
| TSA 1X; TSA 0.1X; TSA 0.01X; R2A 1X; R2A 0.01X | 1 | Kocuria |
| TSA 1X; TSA 0.1X; TSA 0.01X; R2A 0.1X; R2A 0.01X | 1 | Kineococcus |
| TSA 1X; TSA 0.01X R2A 1X; R2A 0.1X; R2A 0.01X | 1 | Mycolicibacterium |
| TSA 1X; TSA 0.1X; TSA 0.01X; R2A 1X | 5 | Lentzea, Sphingomonas, Roseomonas, Belnapia, Ramlibacter |
| TSA 1X; TSA 0.1X R2A 1X; R2A 0.1X | 1 | Skermanella |
| TSA 1X; TSA 0.01X; R2A 1X | 1 | Modestobacter |
| TSA 0.1X; TSA 0.01X R2A 1X; R2A 0.1X | 1 | Streptomyces |
| TSA 0.1X; R2A 1X; R2A 0.01X | 1 | Methylobacterium |
| TSA 1X; TSA 0.01X | 1 | Planctomonas |
| TSA 0.1X; TSA 0.01X | 1 | Microvirga |
| TSA 0.1X; R2A 1X | 3 | Nocardioides, Geodermatophilus, Umezawaea |
| TSA 0.01X; R2A 1X | 1 | Kribbella |
| TSA 0.01X; R2A 0.01X | 1 | Actinoplanes |
| R2A 1X; R2A 0.1X | 1 | Massilia |
| TSA 1X | 2 | Micrococcus, Staphylococcus |
| TSA 0.1X | 4 | Variovorax, Noviherbaspirillum, Paenarthrobacter, Amycolatopsis |
| TSA 0.01X | 2 | Enterovirga, New genus |
| R2A 1X | 4 | Agrococcus, Blastococcus, Caulobacter, Azospirillum |
| R2A 0.1X | 2 | Herbaspirillum, Bacillus |
| R2A 0.01X | 1 | Flaviflagellibacter |

## Slide 2
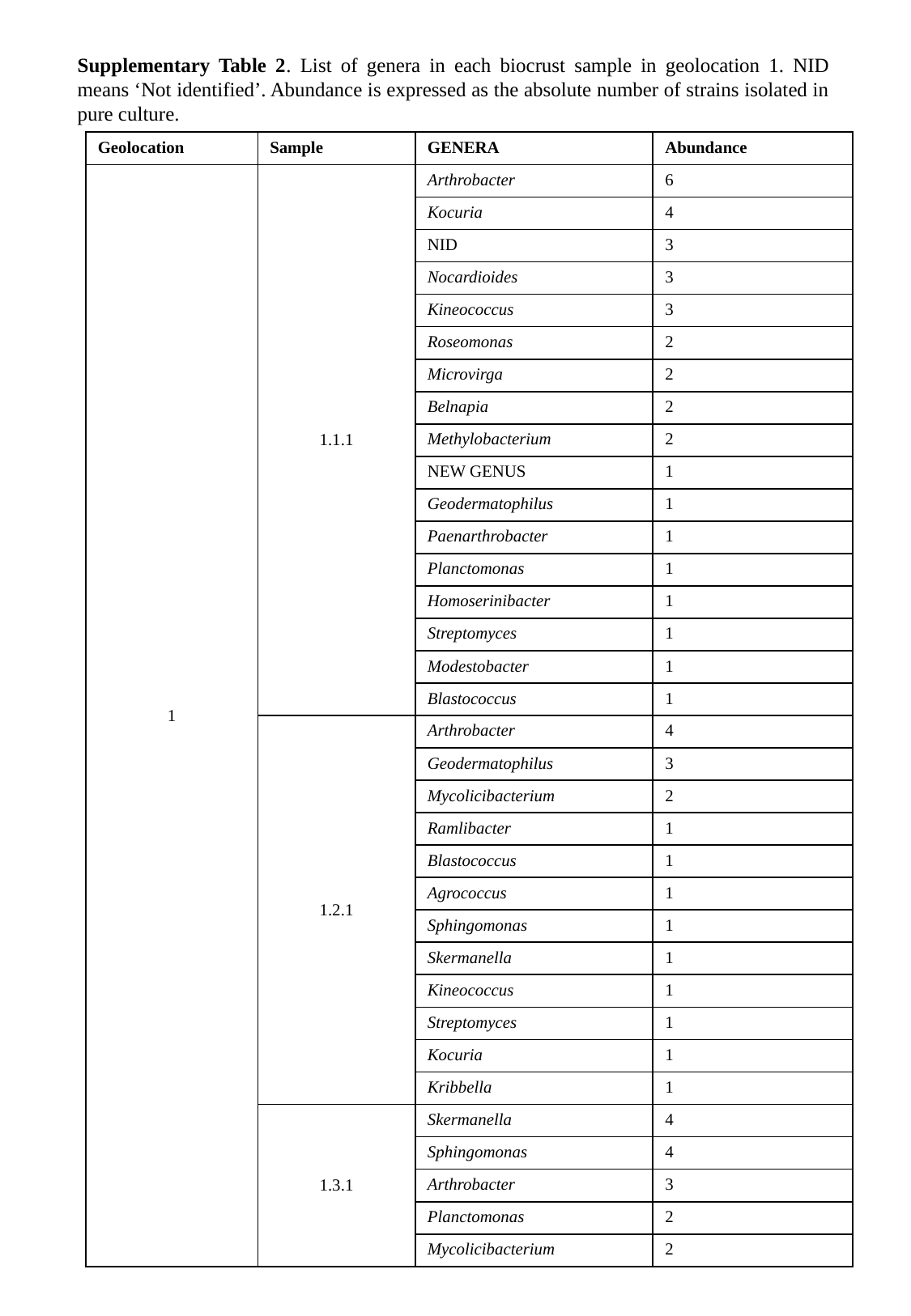

Supplementary Table 2. List of genera in each biocrust sample in geolocation 1. NID means ‘Not identified’. Abundance is expressed as the absolute number of strains isolated in pure culture.
| Geolocation | Sample | GENERA | Abundance |
| --- | --- | --- | --- |
| 1 | 1.1.1 | Arthrobacter | 6 |
| | | Kocuria | 4 |
| | | NID | 3 |
| | | Nocardioides | 3 |
| | | Kineococcus | 3 |
| | | Roseomonas | 2 |
| | | Microvirga | 2 |
| | | Belnapia | 2 |
| | | Methylobacterium | 2 |
| | | NEW GENUS | 1 |
| | | Geodermatophilus | 1 |
| | | Paenarthrobacter | 1 |
| | | Planctomonas | 1 |
| | | Homoserinibacter | 1 |
| | | Streptomyces | 1 |
| | | Modestobacter | 1 |
| | | Blastococcus | 1 |
| | 1.2.1 | Arthrobacter | 4 |
| | | Geodermatophilus | 3 |
| | | Mycolicibacterium | 2 |
| | | Ramlibacter | 1 |
| | | Blastococcus | 1 |
| | | Agrococcus | 1 |
| | | Sphingomonas | 1 |
| | | Skermanella | 1 |
| | | Kineococcus | 1 |
| | | Streptomyces | 1 |
| | | Kocuria | 1 |
| | | Kribbella | 1 |
| | 1.3.1 | Skermanella | 4 |
| | | Sphingomonas | 4 |
| | | Arthrobacter | 3 |
| | | Planctomonas | 2 |
| | | Mycolicibacterium | 2 |

## Slide 3
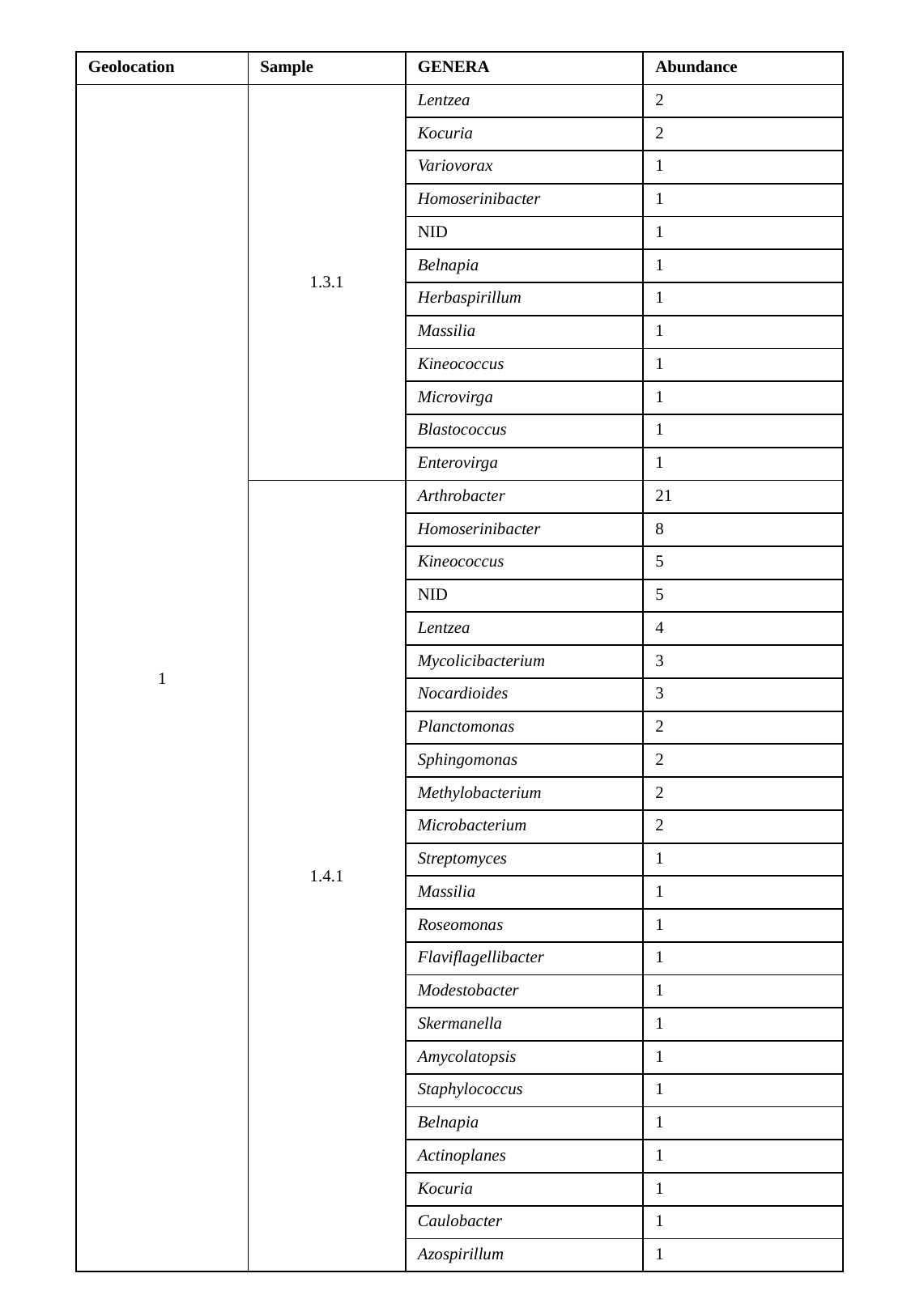

| Geolocation | Sample | GENERA | Abundance |
| --- | --- | --- | --- |
| 1 | 1.3.1 | Lentzea | 2 |
| | | Kocuria | 2 |
| | | Variovorax | 1 |
| | | Homoserinibacter | 1 |
| | | NID | 1 |
| | | Belnapia | 1 |
| | | Herbaspirillum | 1 |
| | | Massilia | 1 |
| | | Kineococcus | 1 |
| | | Microvirga | 1 |
| | | Blastococcus | 1 |
| | | Enterovirga | 1 |
| | 1.4.1 | Arthrobacter | 21 |
| | | Homoserinibacter | 8 |
| | | Kineococcus | 5 |
| | | NID | 5 |
| | | Lentzea | 4 |
| | | Mycolicibacterium | 3 |
| | | Nocardioides | 3 |
| | | Planctomonas | 2 |
| | | Sphingomonas | 2 |
| | | Methylobacterium | 2 |
| | | Microbacterium | 2 |
| | | Streptomyces | 1 |
| | | Massilia | 1 |
| | | Roseomonas | 1 |
| | | Flaviflagellibacter | 1 |
| | | Modestobacter | 1 |
| | | Skermanella | 1 |
| | | Amycolatopsis | 1 |
| | | Staphylococcus | 1 |
| | | Belnapia | 1 |
| | | Actinoplanes | 1 |
| | | Kocuria | 1 |
| | | Caulobacter | 1 |
| | | Azospirillum | 1 |

## Slide 4
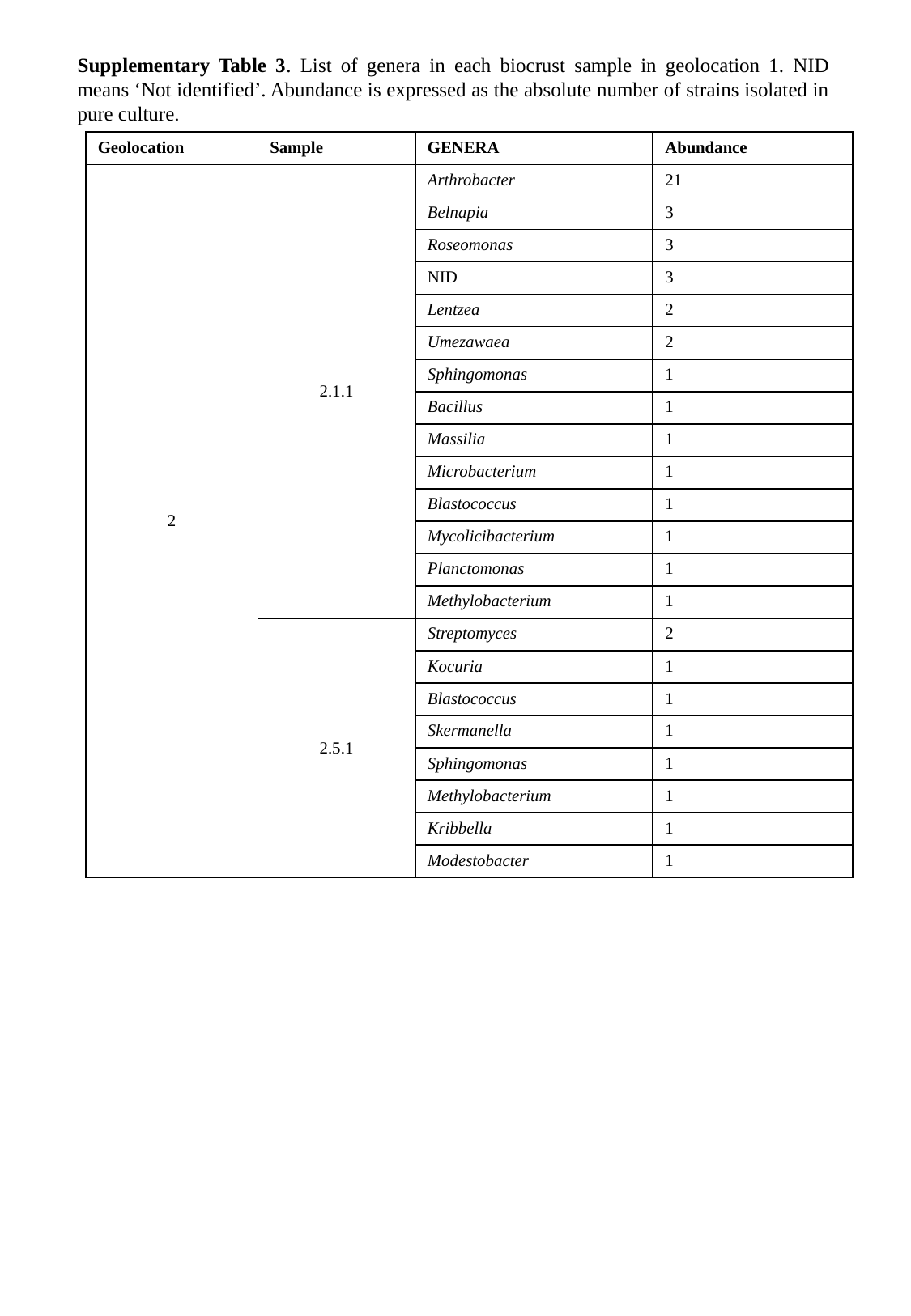

Supplementary Table 3. List of genera in each biocrust sample in geolocation 1. NID means ‘Not identified’. Abundance is expressed as the absolute number of strains isolated in pure culture.
| Geolocation | Sample | GENERA | Abundance |
| --- | --- | --- | --- |
| 2 | 2.1.1 | Arthrobacter | 21 |
| | | Belnapia | 3 |
| | | Roseomonas | 3 |
| | | NID | 3 |
| | | Lentzea | 2 |
| | | Umezawaea | 2 |
| | | Sphingomonas | 1 |
| | | Bacillus | 1 |
| | | Massilia | 1 |
| | | Microbacterium | 1 |
| | | Blastococcus | 1 |
| | | Mycolicibacterium | 1 |
| | | Planctomonas | 1 |
| | | Methylobacterium | 1 |
| | 2.5.1 | Streptomyces | 2 |
| | | Kocuria | 1 |
| | | Blastococcus | 1 |
| | | Skermanella | 1 |
| | | Sphingomonas | 1 |
| | | Methylobacterium | 1 |
| | | Kribbella | 1 |
| | | Modestobacter | 1 |

## Slide 5
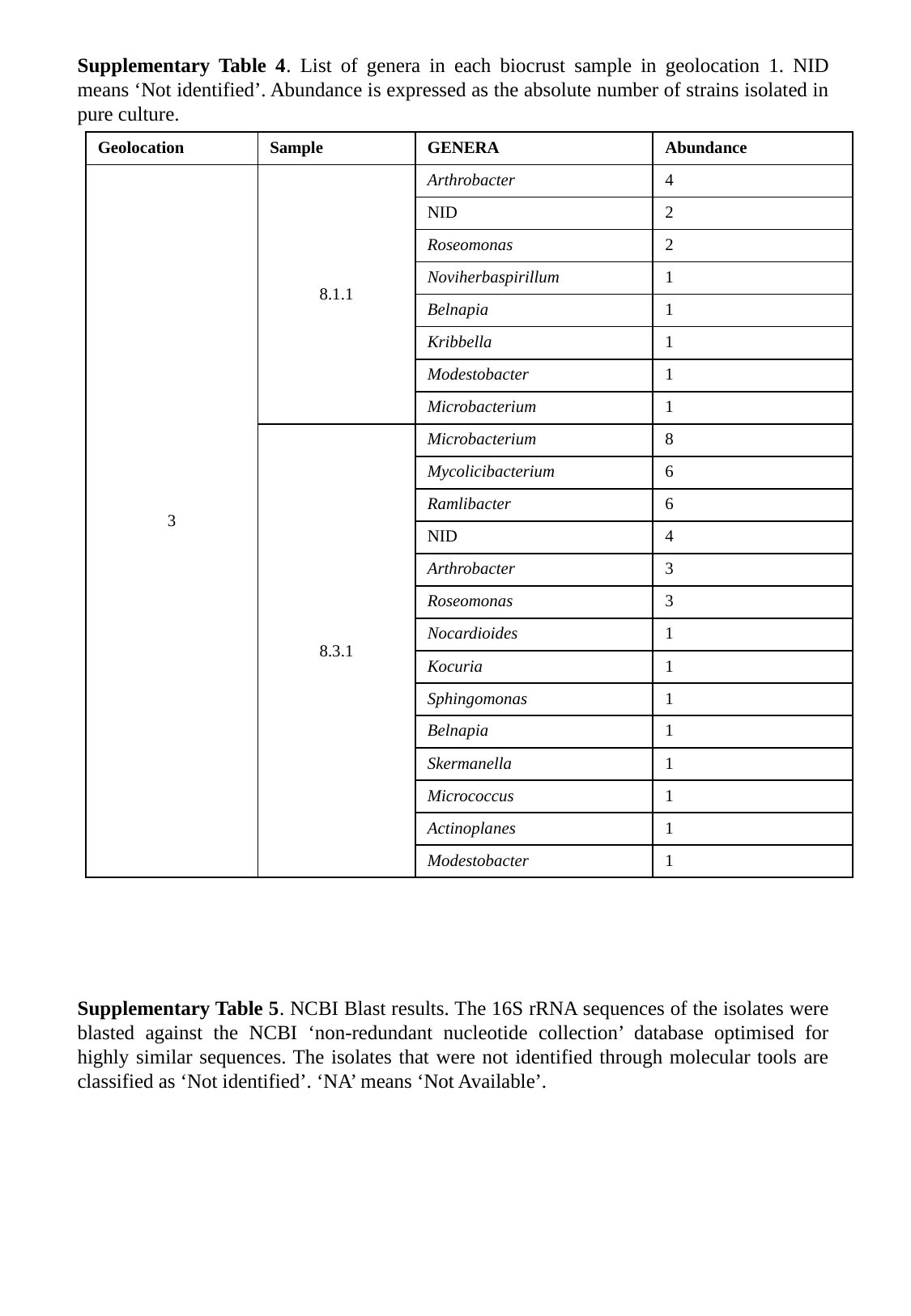

Supplementary Table 4. List of genera in each biocrust sample in geolocation 1. NID means ‘Not identified’. Abundance is expressed as the absolute number of strains isolated in pure culture.
| Geolocation | Sample | GENERA | Abundance |
| --- | --- | --- | --- |
| 3 | 8.1.1 | Arthrobacter | 4 |
| | | NID | 2 |
| | | Roseomonas | 2 |
| | | Noviherbaspirillum | 1 |
| | | Belnapia | 1 |
| | | Kribbella | 1 |
| | | Modestobacter | 1 |
| | | Microbacterium | 1 |
| | 8.3.1 | Microbacterium | 8 |
| | | Mycolicibacterium | 6 |
| | | Ramlibacter | 6 |
| | | NID | 4 |
| | | Arthrobacter | 3 |
| | | Roseomonas | 3 |
| | | Nocardioides | 1 |
| | | Kocuria | 1 |
| | | Sphingomonas | 1 |
| | | Belnapia | 1 |
| | | Skermanella | 1 |
| | | Micrococcus | 1 |
| | | Actinoplanes | 1 |
| | | Modestobacter | 1 |
Supplementary Table 5. NCBI Blast results. The 16S rRNA sequences of the isolates were blasted against the NCBI ‘non-redundant nucleotide collection’ database optimised for highly similar sequences. The isolates that were not identified through molecular tools are classified as ‘Not identified’. ‘NA’ means ‘Not Available’.
